# A somatosensory fovea in the fingertips of the Surinam toad

**DOI:** 10.1007/s00359-026-01838-w

**Published:** 2026-08-10

**Authors:** Christopher J. Meredith, Rebeca W. Fuquen, Sophia Manfrey, Penelope Chai, Duncan B. Leitch

**Affiliations:** Department of Integrative Biology and Physiology, University of California Los Angeles, Los Angeles, CA 90095 USA

**Keywords:** Mechanoreception, Somatosensory fovea, Cortical magnification, Optic tectum, *Pipa pipa*, Suction feeding

## Abstract

The Surinam toad (*Pipa pipa*) is a fully aquatic, tongueless pipid frog that captures prey using rapid suction feeding coordinated by the forelimbs. The digit tips bear distinctive lobed structures whose function has been unknown. Here we characterize the morphology, mechanical sensitivity, peripheral innervation, central representation, and behavioral function of these digit tip lobes using scanning electron microscopy, von Frey threshold measurements, in vivo electrophysiology of the brachial nerve and optic tectum, and high-speed video analysis (1000 fps). SEM reveals a progressive quadrification yielding 128 terminal lobules per frog, bearing dome-shaped papillae at 142 ± 18/mm^2^—nearly fourfold the density on adjacent digit shaft skin. Distal lobule tips have the lowest mechanical thresholds on the forelimb (median 0.07 mN). Brachial nerve recordings reveal disproportionately represented, low-threshold, predominantly rapidly adapting mechanoreceptive afferents with small receptive fields confined to the lobules, some also responsive to water current stimulation. Tectal mapping shows the digit tips are somatotopically represented and disproportionately magnified, occupying 34% of the forelimb representation despite comprising only 8% of forelimb surface area (magnification factor: 4.3×). High-speed behavioral recordings under visible light, infrared, and cobalt-blocked lateral line conditions demonstrate that frogs initiate suction feeding before prey fish contact the digits, indicating detection of hydrodynamic disturbances at short range (~ 5 mm). These results establish the digit tip lobes of *Pipa pipa* as somatosensory foveae—specialized mechanoreceptive organs analogous to the tactile fovea of the star-nosed mole.

**Supplementary Information:**

The online version contains supplementary material available at https://doi.org/10.1007/s00359-026-01838-w.

## Introduction

Across vertebrate sensory systems, a recurring organizational theme is the concentration of receptors and neural processing resources in a small, behaviorally critical region of the sensory surface—a sensory fovea. Originally described for the primate retina, where cone photoreceptor density peaks sharply at the center of gaze and the corresponding cortical representation is disproportionately expanded (Azzopardi and Cowey 1993), the foveal concept has been extended to the acoustic system of FM-CF echolocating bats (Suga et al. 1987; Covey 2005), the electrosensory bill of the platypus (Manger and Pettigrew 1996; Pettigrew 1999), the bill tips of probing shorebirds (Pettigrew and Frost 1985; Cunningham et al. 2010), and, most thoroughly, to the somatosensory star of the star-nosed mole (*Condylura cristata* Catania and Kaas 1997a; Catania and Remple 2004; Catania et al. 2011). In each case, three defining features are present: (1) a region of elevated receptor density or innervation, (2) a disproportionately large central representation relative to the sensory surface area, and (3) a sensorimotor strategy that actively repositions the foveal region onto stimuli of interest. In this study, we use “somatosensory fovea” in the strict operational sense, satisfying all three criteria jointly and distinguish it from “tactile magnification” (criterion 2 alone, satisfied by, e.g., the primate hand and the lips in the cortical homunculus) and “tactile specialization” (criterion 1 alone). Additional mammalian examples include the trigeminally innervated tip of the African elephant trunk, where arrays of vibrissa-bearing finger-like protrusions support remarkable somatosensory acuity, dexterous object manipulation, and a disproportionate representation in trigeminal nuclei (Purkart et al. 2022; Deiringer et al. 2023; Longren et al. 2023; Schulz et al. 2025). Although tactile foveae have been proposed for the bill tips of probing shorebirds (Pettigrew and Frost 1985; Cunningham et al. 2010) on the basis of receptor density and behavioral evidence, no non-mammalian system has been characterized using the full three-criterion framework, including direct demonstration of central magnification.

The star-nosed mole provides the most complete model of a somatosensory fovea. Its 22 fleshy nasal appendages are covered with approximately 25,000 Eimer’s organs, each containing Merkel cell–neurite complexes, lamellated corpuscles, and free nerve endings (Catania 1995). The 11th (ventromedial) appendage, though small and bearing relatively few Eimer’s organs, has the greatest innervation density per organ, the largest cortical representation in S1, and is preferentially repositioned onto objects of interest via saccade-like movements during foraging (Catania and Kaas 1997b). The degree of cortical over-representation (approximately three- to fourfold greater area of cortex per afferent for appendage 11 than for peripheral appendage) are termed “afferent magnification,” emphasizing that the neural over-representation exceeds what would be predicted from innervation density alone (Catania and Remple 2004). Subsequent work traced the magnification cascade from the peripheral nerve through the principal sensory nucleus (PrV) (Catania et al. 2011) and spinal trigeminal nuclei of the brainstem (Sawyer et al. 2014) to the superior colliculus (SC; Crish et al. 2003) to S1, revealing that magnification increases at each successive relay. These findings established a quantitative framework for identifying somatosensory foveae that we apply here to a non-mammalian system.

The Surinam toad (*Pipa pipa*), the largest member of the fully aquatic frog family Pipidae, is an obligate aquatic species native to the slow-moving, tannin-rich waters of the Amazon and Orinoco basins. Like all pipids, *P. pipa* is tongueless and relies on rapid suction feeding to capture prey (Carreño and Nishikawa 2010; Fernandez et al. 2017). During prey capture, the forelimbs are held in a forward-flexed posture anterior to the head and either directly guide prey into the expanding buccal cavity or guard against escape during the suction event. *P. pipa* is capable of enlarging its entire trunk during suction feeding, achieving buccal expansion in 12–24 ms (Fernandez et al. 2017). The digit tips terminate in distinctive lobed or “star-shaped” structures that have been noted in taxonomic descriptions (Cannatella and Trueb 1986) but whose function has never been experimentally investigated.

The family Pipidae occupies a basal position among neobatrachian frogs and represents a radiation of secondarily fully aquatic species that diverged in the late Mesozoic, predating the breakup of Gondwana (Cannatella 2015). The family is divided into two subfamilies: Pipinae (*Pipa*, South America) and Xenopodinae (*Xenopus*, *Hymenochirus*, *Silurana*, Africa). All pipids are fully aquatic, but they exhibit a pronounced gradient of digit tip elaboration: *Xenopus* species have simple conical digit tips with no lobing; *Hymenochirus* has modestly lobed tips; and *Pipa* species have the most elaborate structures, with *P. pipa* displaying the most pronounced quadrification (Trueb 1984; Cannatella and Trueb 1986). This morphological gradient, together with the observation that *Pipa* species inhabit the most turbid, visually-impoverished habitats among pipids, raises the hypothesis that digit tip elaboration is an adaptation for enhanced mechanosensory prey detection in environments where vision is unreliable.

The habitat and foraging ecology of *P. pipa* impose sensory challenges that are instructive to consider. The tannin-stained, often sediment-laden waters of its native range considerably limit visual range. *P. pipa* has small, dorsally directed, lidless eyes with limited binocular overlap—an arrangement poorly suited for precise visual prey localization at close range in murky water. While the lateral line system of *Pipa pipa* is presumably functional (all pipids retain neuromasts) and lateral-line input supports impressive prey localization in many fishes (Coombs and Montgomery 1999; Bleckmann and Zelick 2009), the spatial resolution of the pipid lateral line has not been systematically characterized and may be limited by the relatively sparse neuromast distribution in this family. We hypothesize that the digit tip lobes serve as a high-resolution, short-range mechanosensory system that fills the gap between coarse lateral line detection at distance and the final sensorimotor decision to initiate suction feeding.

A critical feature of our study is that we investigate central representation of the digit tips in the optic tectum rather than in the neocortex, as amphibians lack a neocortex. In anurans, the tectum (homolog of the mammalian SC) is a key integrative center for spatial sensory processing and sensorimotor transformation; it receives topographically organized visual input (Lázár 1984) and, in the deep layers, somatosensory afferents arising in part from dorsal-column and trigeminal projections (Lowe 1986; Muñoz et al. 1995). The torus semicircularis, primarily the auditory midbrain nucleus in anurans, has also been reported to receive somatosensory input, predominantly with broad receptive fields restricted to its lateral subdivision (Comer and Grobstein 1981a; Muñoz et al. 1997); we return to the tectum/torus distinction in the Discussion. The tectum plays a role in anuran prey capture behavior, analogous to that of the SC in mammalian orienting responses (Ingle 1973; Ewert 2004). Demonstrating somatosensory foveal magnification in the tectum would extend the principle of foveal organization to a brain structure phylogenetically older than the mammalian neocortex.

Here, we combine scanning electron microscopy, calibrated mechanical threshold measurements, in vivo single- and multi-unit electrophysiology of the brachial nerve and optic tectum, and high-speed video recording of suction feeding behavior under multiple sensory-deprivation conditions to provide a comprehensive neuroethological characterization of the digit tip lobes of *Pipa pipa*. Our results demonstrate that these structures meet all three criteria for a somatosensory fovea and reveal a remarkable functional and organizational convergence with the nasal appendages of the star-nosed mole.

In the context of this neurobiological puzzle, requiring complementary neurophysiological and behavioral perspectives towards unraveling the function of novel sensory adaptation, this study is dedicated to Peter Narins. His decades of work on amphibian sensory neurobiology have shaped how we think about how anurans extract behaviorally critical information from physically rich environments. Narins and colleagues established many of the foundational principles of anuran multimodal integration, from the acoustic and seismic-vibrational communication of frogs (Narins and Capranica 1976; Lewis and Narins 1985; Narins 1990), to the high-precision detection of substrate-borne and near-field hydrodynamic signals (Christensen-Dalsgaard and Narins 1993; Narins et al. 2005), to the comparative neuroethology of prey-relevant signal extraction. The recurring lesson of this work—that anurans are exquisitely tuned to the physical structure of their habitats, often through specialized peripheral organs whose central representation reflects their behavioral importance—is the conceptual scaffolding on which the present study is built.

## Materials and methods

### Animals

Adult and juvenile *Pipa pipa* (*n* = 19 total; 8 adults, snout–vent length [SVL] 105–156 mm; 11 juveniles, SVL 82–98 mm) were obtained from licensed commercial suppliers and maintained in glass aquaria (75 L) filled with dechlorinated, carbon-filtered water at 26–28 °C on a 12:12 h light: dark cycle. Water was maintained at pH 6.0–7.2 with a pH-lowering reagent (API pH Down; Mars Fishcare, Chalfont, PA) and Indian almond leaf extract to approximate natural habitat conditions. Animals were fed live minnows (*Pimephales promelas*), goldfish (*Carassius auratus*), blackworms (*Lumbriculus variegatus*), and frozen bloodworms (*Chironomus* spp.) three times weekly. Animals were acclimated for a minimum of two weeks before experiments. Several animals contributed to more than one procedure (with at least 4 weeks between procedures, where applicable). All procedures were approved by the UCLA Institutional Animal Care and Use Committee (protocol #ARC 2024-025) and conducted in accordance with the National Institutes of Health Guide for the Care and Use of Laboratory Animals. All animals had intact tympanic disks and middle-ear apparatus throughout the study; no middle-ear, otolith, or VIIIth-nerve manipulations were performed.

### Scanning electron microscopy

Forelimb digits (*n* = 10 digits from 2 adults, 12 digits from 2 juveniles) were dissected at the metacarpophalangeal joint from euthanized frogs (0.5% tricaine methanesulfonate [MS-222; Sigma-Aldrich, St. Louis, MO] immersion, pH 7.0, followed by decapitation), fixed in 4% paraformaldehyde in 0.1 M sodium phosphate buffer (pH 7.4) for > 24 h, and rinsed (3 × 15 min in PBS). Specimens were dehydrated through a graded ethanol series (30%, 50%, 70%, 80%, 90%, 95%, 100%, 100%; >15 min each), critical-point dried using liquid CO_2_, mounted on aluminum stubs with carbon tape, sputter-coated with 15 nm gold-palladium alloy, and imaged on a FEI Quanta 650 field-emission scanning electron microscope at accelerating voltages of 5–15 kV in the UCLA California Nanosystems Institute.

Papillae density was quantified from calibrated SEM micrographs using ImageJ (v1.54, NIH). For each digit examined, four non-overlapping 200 × 200 μm quadrats were placed randomly on each of four surfaces: the distal lobule tip (the most distal surface of the secondary lobule), the proximal lobule base, the mid-digit shaft, and the inter-digital margin. Individual dome-shaped papillae were counted within each quadrat. Papillae diameter was measured from the base of 20 randomly selected papillae per surface per digit. All measurements were performed by a single observer.

### Von Frey threshold measurements

Mechanical thresholds were measured in 3 adult frogs using calibrated von Frey monofilaments (Stoelting Touch-Test Sensory Evaluator kit; range 0.008–300 mN). Frogs were anesthetized by immersion in 0.1% MS-222 buffered to pH 7.0 with sodium bicarbonate until withdrawal reflexes were abolished (typically 15–20 min). Anesthesia was maintained throughout the procedure by wrapping the trunk in gauze saturated with 0.05% MS-222 solution, leaving the test forelimb exposed in a shallow dish of dechlorinated water.

Five standardized test locations were defined on each digit of both forelimbs: (1) the distal lobule tip (center of the most distal secondary lobule), (2) the proximal lobule base (junction between the primary lobe and the digit shaft), (3) the mid-digit shaft (midpoint between the proximal lobule base and the metacarpophalangeal joint), (4) the inter-digital webbing margin (midpoint of the web between adjacent digits), and (5) the metacarpal pad (center of the ventral palm). At each location, filaments were applied perpendicular to the skin surface with sufficient force to cause the filament to bend (ensuring consistent force delivery), held for approximately 1 s, and removed. Testing proceeded in ascending order from the finest filament. A “positive response” was defined as any detectable withdrawal movement of the digit or limb, assessed under a dissecting microscope at 10× magnification. Subsequently, the brachial nerve was simultaneously recorded (see below) to verify that behavioral thresholds corresponded to afferent activation thresholds. Each location was tested three times with each filament; threshold was defined as the lowest force filament eliciting a positive response in at least two of three applications. Test locations were randomized within each session to prevent order effects.

### In vivo electrophysiology: brachial nerve

Single- and multi-unit extracellular recordings were obtained from the brachial nerve of 6 adult frogs. Animals were anesthetized with MS-222 as described above, supplemented with intramuscular xylazine (4 mg/kg body mass) as needed to maintain a stable anesthetic plane. The frog was placed ventral side up on a custom platform within a small dish, and the forelimb was secured in a semi-extended position with insect pins through the metacarpal region (pins placed to avoid damage to the digits and digital nerves). The brachial nerve was exposed via a longitudinal incision (5–8 mm) through the ventral skin and pectoral musculature proximal to the shoulder, and the nerve was supported on a small platform of dental silicone (Reprosil, Dentsply Sirona) for stability. We recorded from the main mixed brachial-nerve trunk proximal to its branching into musculocutaneous, radial, and median subdivisions; this trunk carries cutaneous afferents from the entire forelimb.

Extracellular recordings were made with tungsten microelectrodes (FHC Inc., Bowdoin, ME; impedance 5–12 MΩ at 1 kHz), advanced into the nerve using a hydraulic micromanipulator (Sutter SOLO) mounted on a magnetic stand. A chlorided silver reference electrode was placed in nearby muscle tissue. Signals were amplified 10,000× (A-M Systems Model 1800 differential amplifier; bandpass 300 Hz–10 kHz), digitized at 40 kHz (Cambridge Electronic Design [CED] Micro1401-3), and recorded using Spike2 software (v10, CED) or Powerlab (AD Instruments). Audio monitoring (AM Systems AM10) facilitated real-time identification of responsive units.

Receptive fields were mapped by systematically probing the forelimb surface with a blunt glass probe (tip diameter ~ 100 μm, fashioned from borosilicate capillary tubing or wood) and calibrated von Frey filaments. For each isolated unit, the receptive field boundary was defined as the region within which stimulation with the 0.4 mN filament evoked a response; this boundary was drawn on a scaled photograph of the forelimb taken at the beginning of each session. Mechanical threshold at the receptive field center was determined with von Frey filaments as described above. Response adaptation was classified by applying a suprathreshold von Frey filament (1.0 mN for digit tip units, 4.0 mN for digit shaft units) for 5 s: rapidly adapting (RA) units responded only at onset and offset; slowly adapting (SA) units maintained firing throughout the stimulus.

To test sensitivity to hydrodynamic stimuli, a gravity-fed laminar flow system was used to deliver water currents across the digit tips. Dechlorinated water at 27 °C was fed from an elevated reservoir through polyethylene tubing (inner diameter 2 mm), with flow velocity (range: 2–15 cm/s) controlled by adjusting reservoir height and calibrated with a micro-flowmeter (Sensirion SLQ-QT500). The tube outlet was positioned 5 mm from the digit tips, oriented to direct flow parallel to the long axis of the digit. For non-digit recording sites (digit shaft, palm, forearm), the flow tube was repositioned so that the outlet was 5 mm from the receptive-field center and oriented perpendicular to the local skin surface to maximize displacement-axis stimulation. In a subset of 12 non-digit units we additionally tested four flow orientations (0°, 45°, 90°, and 135° relative to the local skin axis) at 6 cm/s; none evoked detectable responses. Flow onset was controlled with a solenoid valve (Parker Hannifin) triggered by the Spike2 data acquisition system, enabling precise temporal alignment between stimulus and neural recording. Spike sorting was performed offline in Spike2 using template matching followed by principal component analysis (PCA)-based cluster separation. Only units with a signal-to-noise ratio > 3:1 and a clean refractory period in the interspike interval histogram (ISI < 1.5 ms events < 1% of total) were included in subsequent analyses.

### In vivo electrophysiology: optic tectum

Systematic multi-unit mapping of the contralateral optic tectum was performed in 2 adult and 3 juvenile frogs. Anesthesia was induced and maintained as for brachial nerve recordings. The frog was placed in a custom head-holding apparatus that stabilized the skull via a moveable joint while leaving the contralateral forelimb freely accessible. A craniotomy (approximately 3 × 4 mm) was made over the dorsal tectum by thinning the parietal bone with a dental drill under saline irrigation and removing the thinned bone with fine forceps. The dura was left intact where possible, or incised with a fine needle where it impeded electrode penetration. The exposed tectum was kept moist with Ringer’s solution (NaCl 111 mM, KCl 2.5 mM, CaCl_2_ 1.8 mM, MgCl_2_ 1.0 mM, glucose 11 mM, HEPES 5 mM, pH 7.4).

Multi-unit extracellular recordings were made with tungsten microelectrodes (FHC, 1–5 MΩ), advanced perpendicular to the tectal surface with the hydraulic micromanipulator. Electrode penetrations were made roughly following a grid with 200 μm spacing across the accessible tectal surface (typical grid: 15–20 mediolateral × 20–25 rostrocaudal positions, for a total of about 120 penetrations per animal). At each site, the electrode was advanced to a depth of 200–500 μm below the tectal surface, targeting the deep layers where somatosensory afferents terminate (Lázár 1984). Somatosensory receptive fields were mapped by systematically probing the contralateral forelimb surface with calibrated von Frey filaments and a blunt glass probe. For sites responsive to forelimb stimulation, the receptive field center and boundaries were drawn on a standardized photograph of the forelimb. At each responsive site, minimum threshold was determined with von Frey filaments as described above. After completion of the forelimb map at each penetration, we additionally probed the contralateral hindlimb, dorsal head, ventral head, and trunk to identify the spatial extent of the forelimb representation and to provide the multi-region overview shown in Fig. 4h. Receptive-field tracing for these non-forelimb regions used the same von Frey + glass-probe approach as for the forelimb.

Following physiological recording, electrolytic lesions (10 µA for 5–10 s) were made within tactile-responsive tectal layers (Ugo Basile Lesion Making Device). Following transcardial perfusion with PBS followed by 4% paraformaldehyde in 0.1 M sodium phosphate buffer (pH 7.4), brains were dissected, post-fixed in the same fixative for 24 h at 4 °C, cryoprotected for > 24 h in 30% sucrose in PBS, sectioned at 50 μm on a freezing microtome, and stained for thionin (Windle et al. 1943).

Somatotopic maps were reconstructed by assigning each tectal recording site a color code corresponding to the forelimb region at the center of its receptive field (digit tips, digit shafts, palm, or forearm). The tectal area devoted to each forelimb region was reconstructed by aligning photographs of the craniotomy and recorded penetrations to the midline. The proportional area of the tectum devoted to each forelimb region was then calculated. Magnification factors were computed as the ratio of the proportional tectal area to the proportional peripheral surface area for each region. Peripheral surface areas were measured from calibrated photographs of the forelimb using ImageJ.

Receptive field areas were measured from the traced outlines on standardized forelimb photographs using ImageJ. Where a single recording site yielded responses to multiple non-contiguous skin regions (9/216 sites yielded two non-contiguous responses; 1/216 yielded three), only the primary (lowest-threshold) receptive field was included in the area analysis.

### High-speed behavioral recordings

Suction feeding behavior directed at live prey was recorded in 2 adult and 5 juvenile frogs using high-speed video. Recordings were made in a custom glass aquarium (40 × 20 × 20 cm) filled to a depth of 10 cm with dechlorinated water at 27 °C. A Chronos 4K12 high-speed camera fitted with a Nikon AF Micro-Nikkor 105 mm f/2.8D lens was positioned lateral to the aquarium, recording at 1000 frames per second (fps) with a spatial resolution of approximately 50 μm/pixel at the focal plane of the frog’s forelimb.

Three illumination and pharmacological conditions were tested, each designed to selectively eliminate one or more sensory modalities:

*Visible light condition.* Overhead and tangential LED illumination (400–700 nm, ~ 500 lx at the water surface), providing full visual information to the frog.

*Infrared-only condition.* All visible light was eliminated; the aquarium was illuminated exclusively with an 850–940 nm LED array (Univi), which is outside the spectral sensitivity of anuran photoreceptors (Govardovskii et al. 2000). The high-speed camera’s IR filter was removed to enable imaging. This condition eliminated visual prey detection while leaving all other sensory modalities intact.

*Infrared + cobalt condition.* Infrared illumination as above, combined with the addition of cobalt chloride (CoCl_2_, 0.1 mM; Sigma-Aldrich) to the water. Cobalt ions reversibly block mechanotransduction in lateral line neuromast hair cells by interfering with calcium-dependent transduction channels (Hassan 1985; Karlsen and Sand 1987; Mogdans and Bleckmann 2012), without affecting somatosensory mechanoreceptors at this concentration. This condition eliminated both vision and lateral line function, leaving somatosensory, chemosensory, and olfactory modalities intact.

Each frog was tested 10 times in each condition (210 total feeding events across 7 frogs), with conditions presented in a randomized block design. A minimum 48-h washout period was imposed between cobalt and non-cobalt sessions, during which frogs were maintained in fresh, cobalt-free water. At the start of each trial, a single live fish (*Pimephales*, 15–25 mm total length) was introduced into the behavioral arena. Recording continued until the frog either captured the prey or 600 s had elapsed without a capture attempt.

Videos were analyzed frame-by-frame in ImageJ/Fiji (v2.14.0). Three metrics were extracted from each successful feeding event: (a) the minimum distance between the prey fish body (excluding fins) and the nearest digit surface at the moment of suction initiation, defined as the first frame in which buccal expansion was detectable (initial lateral excursion of the jaw margin); (b) whether direct physical contact between the prey and any digit surface had occurred prior to suction initiation (scored as a binary yes/no); and (c) the latency between the prey fish’s first entry into the “detection envelope” (defined as the three-dimensional volume within 8 mm of any digit tip) and suction initiation. Distances were measured using the known dimensions of the aquarium and a calibration grid imaged at the focal plane following each session.

### Cobalt chloride efficacy control

To verify that the cobalt protocol used in the behavioral experiments selectively blocked lateral-line afferent activity without affecting somatosensory afferents, we performed within-subject control recordings in 2 adult *Pipa pipa* not used in any other experiment. Animals were anesthetized as for brachial-nerve recordings. A tungsten microelectrode (FHC, 5–10 MΩ) was placed under visual guidance into a superficial neuromast nerve branch on the dorsal trunk (immediately caudal to the head; verified by characteristic spontaneous activity and response to a directed water-jet stimulus). A second microelectrode simultaneously recorded from the brachial nerve as described above, with the digit-tip receptive field of a low-threshold mechanoreceptive afferent identified by von Frey indentation. Baseline activity was recorded for 5 min under dechlorinated 27 °C water. The bath was then exchanged for 0.1 mM CoCl_2_ in the same dechlorinated water (identical to the behavioral protocol), and continuous recording continued for 30 min, followed by a 30 min cobalt-free washout. Two stimuli were repeatedly delivered at 2-min intervals throughout: (i) a 0.4 mN von Frey indentation of the identified digit-tip receptive field, and (ii) a 6 cm/s laminar flow stimulus directed across the same digit. Lateral-line afferent firing rate, brachial-nerve spike count to von Frey indentation, and brachial-nerve burst count to flow were quantified in 2 min bins. Statistical comparison of pre-cobalt versus mid-cobalt periods used paired Wilcoxon signed-rank tests.

### Statistical analysis

All statistical analyses were performed in R (v4.3.1, R Foundation for Statistical Computing) or GraphPad Prism (version 9). Data were tested for normality (Shapiro-Wilk test) and homoscedasticity (Levene’s test) before selecting parametric or non-parametric tests. Von Frey threshold data, which were strongly right-skewed, were analyzed with non-parametric tests (Kruskal-Wallis with Dunn’s post-hoc comparisons, Bonferroni-corrected). Receptive field areas and magnification factors, which approximated normality after log-transformation, were analyzed with parametric tests (one-way ANOVA with Tukey’s HSD). Behavioral data (capture success rates, latencies) were analyzed with Friedman’s test (repeated measures, non-parametric) followed by Wilcoxon signed-rank tests with Bonferroni correction. Correlations were assessed with Spearman’s rank correlation coefficient. All values are reported as mean ± standard deviation unless otherwise indicated. Significance was set at α = 0.05 throughout. Effect sizes (Cohen’s *d* for parametric comparisons; rank-biserial correlation *r* for non-parametric comparisons) are reported for all primary analyses.

## Results

### Progressive quadrification of the digit tips yields 128 mechanosensory lobules per frog

**Fig. 1:**
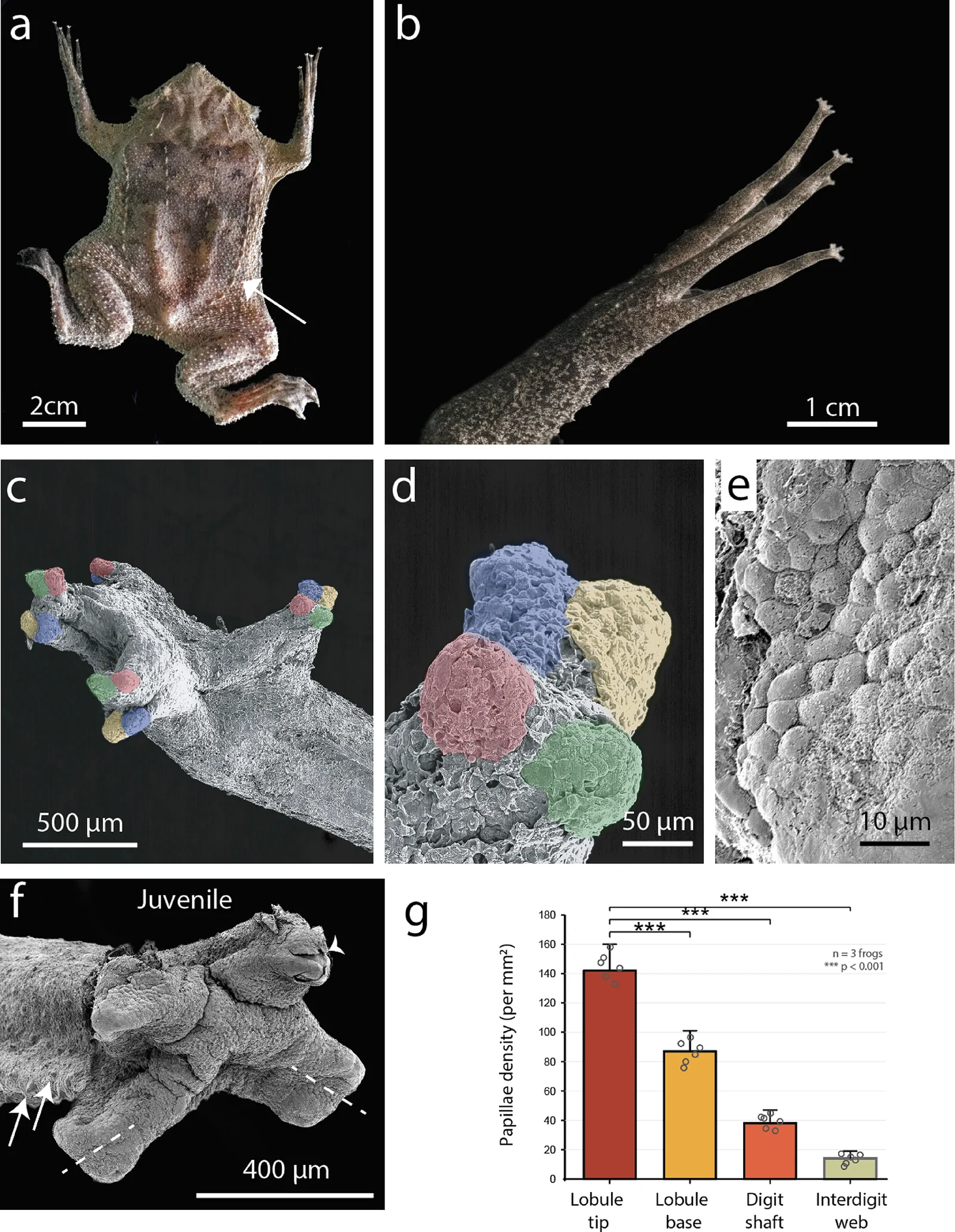
*Pipa pipa* digit tip external morphology. **a** Dorsal view of juvenile *Pipa* with characteristic outstretched forelimbs. Note the prominent lateral line neuromasts (white arrow) also present in adult frogs. Scale bar 2 cm. **b** Higher magnification photograph of the entire forelimb, with the quadrificated tips visible on each of the four digits. Scale bar 1 cm. **c** Scanning electron micrograph (SEM) of a single digit tip from an adult with pseudocolored lobes for clarity. Each of the four primary lobes is quadrificated into another four secondary lobes. Scale bar 500 μm. **d** Single primary lobe showing secondary quadrification into four lobules. Each of the 64 secondary lobes of each forelimb is visible as a dome-shaped structure extending from the digit surface. Scale bar 50 μm. **e** High-magnification view of lobule surface showing dense dome-shaped papillae. Scale bar 10 μm. **f** Digit tip from a juvenile (SVL 88 mm) showing partially fused secondary lobules. The presumptive divisions between secondary lobes are indicated by the dashed lines. Most of the skin is covered by a mucus matrix (arrowhead). Digit tips are covered in domed papillae whereas the digit shaft and proximal regions are covered in prominent, pointed scales (white arrows). Scale bar 400 μm. **g** Papillae density across four forelimb surfaces (*n* = 3 frogs). Open circles indicate individual data points per frog. Error bars: SD

Scanning electron microscopy revealed a stereotyped and hierarchical morphology of the digit tips in adult *Pipa pipa* (Fig. 1a). Each of the four digits on each forelimb terminates in a broad, spatulate tip that is divided into four distinct primary lobes by deep longitudinal sulci extending approximately 400–600 μm proximally from the distal margin (Fig. 1b). Each primary lobe is further subdivided at its distal extremity into four smaller secondary lobules by shallower sulci (depth: 50–80 μm), yielding 16 lobules per digit, 64 per forelimb, and 128 per frog (Fig. 1c, d). The secondary lobules are arranged in a rosette-like pattern, each measuring 180–250 μm in greatest diameter (mean: 212 ± 28 μm, *n* = 96 lobules from 4 frogs). This progressive quadrification pattern, from digit, to four primary lobes, to 16 secondary lobules, was invariant across all specimens examined.

The surfaces of the secondary lobules bear a dense, organized array of dome-shaped papillae (Fig. 1e) often visible through the mucus matrix covering the skin. On the distal (outermost) face of each lobule, papillae density was 142 ± 18 per mm^2^ (*n* = 96 quadrats from 24 lobules, 4 frogs). Papillae density decreased sharply along the proximo-distal axis of the digit: the proximal lobule base bore 87 ± 14 papillae per mm^2^, the mid-digit shaft 38 ± 9 per mm^2^, and the inter-digital webbing margin 14 ± 5 per mm^2^ (one-way ANOVA on log-transformed data, *F*(3,92) = 187.4, *p* < 0.0001; all pairwise comparisons significant at *p* < 0.001, Tukey’s HSD; Fig. 1g). Individual papillae on the lobule tips were 18–25 μm in basal diameter (mean: 21.3 ± 2.7 μm) and approximately 10–15 μm in height, with a smooth, convex surface. These papillae closely resemble the dome-like mechanoreceptive organs described in the integumentary sensory organs (ISOs) of crocodilians (Leitch and Catania 2012) and, to a lesser extent, the Eimer’s organs of the star-nosed mole (Catania 1995), suggesting a mechanosensory function.

In juvenile specimens (SVL < 100 mm), the primary quadrification was well developed, but the secondary lobules were partially fused (Fig. 1f), with the inter-lobule sulci being shallower (< 30 μm) and less distinct. Papillae were present on the digit tips of juveniles but at lower density (94 ± 21 per mm^2^ on distal lobule faces, *n* = 36 quadrats from 2 juveniles). These observations suggest that the full elaboration of the digit tip lobes occurs through post-metamorphic ontogeny, consistent with the protracted post-metamorphic development of other sensory structures in amphibians (Fritzsch 1990).

### Von Frey threshold mapping reveals a steep sensitivity gradient peaking at the digit tips

**Fig. 2:**
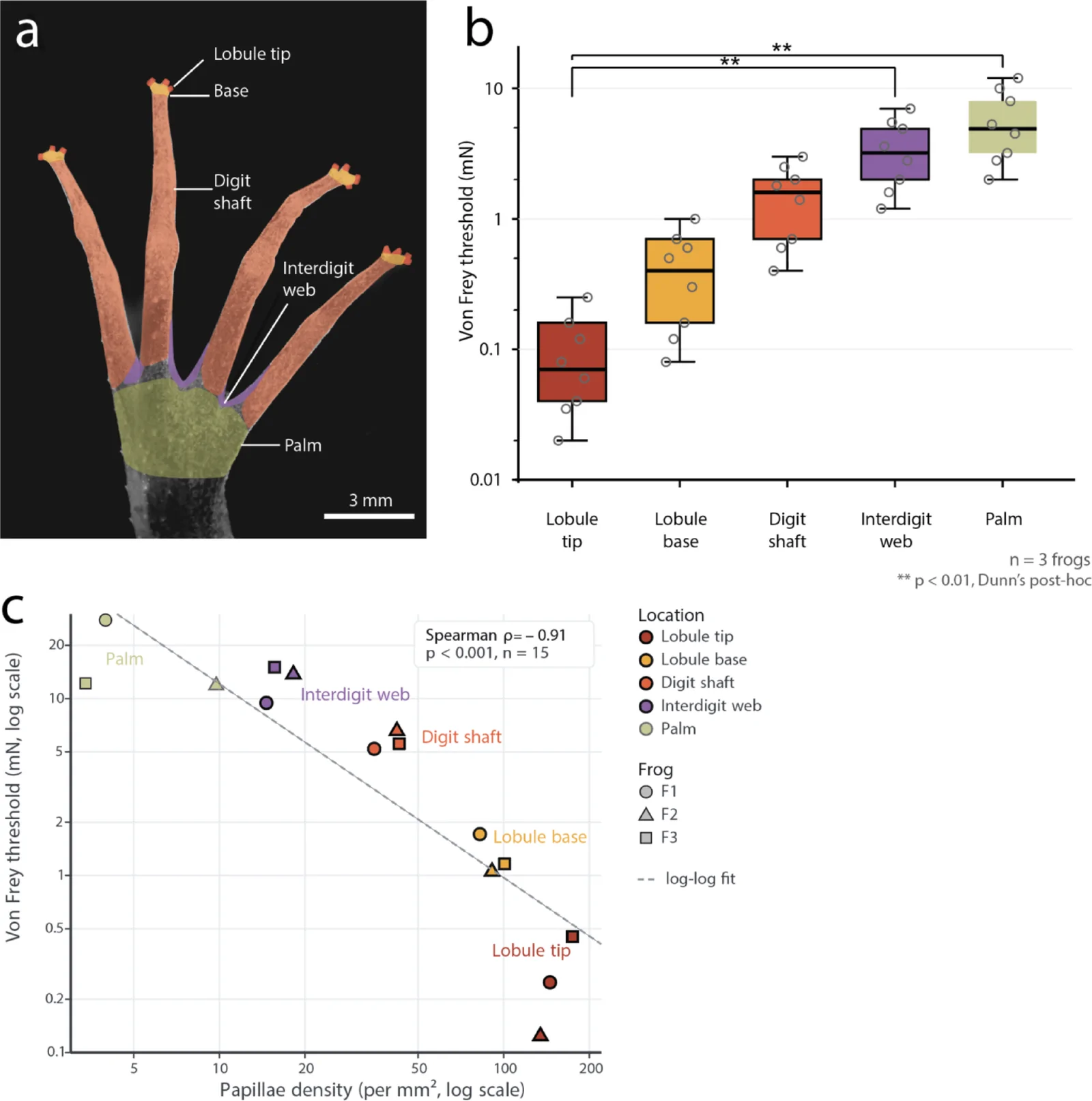
Von Frey threshold mapping of the forelimb. **a** Schematic of the five standardized test locations with regions colored across the dorsal surface. **b** Box-and-whisker plots of thresholds at each location (*n* = 3 frogs; note log scale). Asterisks indicate Dunn’s post-hoc comparisons with lobule tips (**p < 0.01). **c** Threshold versus papillae density across all five locations (Spearman’s *ρ* = −0.91, *p* < 0.001; one point per location per frog)

Mechanical thresholds measured with von Frey filaments across the five standardized forelimb locations revealed a pronounced sensitivity gradient, with the lowest thresholds at the distal lobule tips and the highest at the metacarpal pad (Fig. 2). The median threshold at the distal lobule tips was 0.07 mN (interquartile range [IQR]: 0.04–0.16 mN), compared to 0.4 mN (IQR: 0.16–0.7) at the proximal lobule base, 1.6 mN (IQR: 0.7–2.0) at the mid-digit shaft, 3.2 mN (IQR: 2.0–4.9) at the webbing margin, and 4.9 mN (IQR: 3.2–8.0) at the metacarpal pad (Kruskal-Wallis test, *H* = 47.3, *p* < 0.0001, *n* = 3 frogs; Dunn’s post-hoc comparisons: lobule tip vs. all other locations, *p* < 0.01 for all; rank-biserial *r* > 0.85 for all comparisons involving lobule tips).

The approximately 23-fold difference in threshold between the lobule tips and the mid-digit shaft, and the 70-fold difference between the lobule tips and the metacarpal pad, indicates a remarkably steep sensitivity gradient across a short spatial extent (~ 5–8 mm from lobule tip to palm in an adult). Critically, the sensitivity gradient was strongly correlated with the papillae density gradient measured by SEM: a Spearman’s rank correlation between median von Frey threshold and mean papillae density across the five test locations yielded *ρ* = −0.91 (*p* < 0.001), supporting the interpretation that the dome-shaped papillae are the functional mechanoreceptive units of the digit surface (Fig. 2c).

**Fig. 3:**
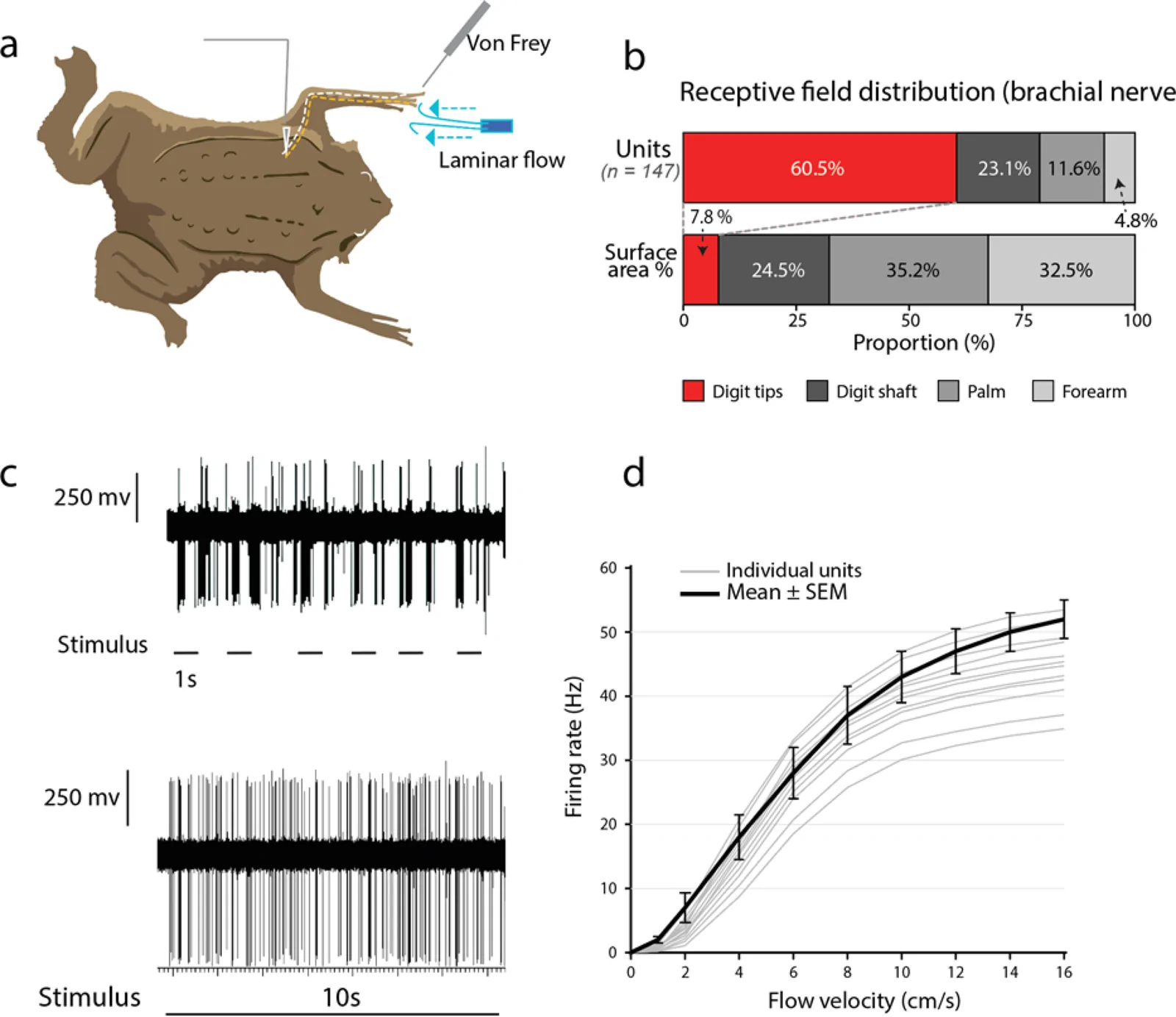
Brachial nerve electrophysiology. **a** Recording preparation schematic with forelimb exposed to mechanical stimulation (von Frey filaments) and laminar flow. **b** Distribution of receptive field locations for 147 units from 6 frogs. Note that 60.5% of units have receptive fields on the digit tips despite this region comprising only ~ 8% of forelimb surface area. **c** Representative single-unit recordings from a rapidly adapting (RA, top) and slowly adapting (SA, bottom) digit tip afferent during von Frey stimulation (stimulus bar below). **d** Firing rate versus flow velocity for 23 flow-sensitive RA units (individual unit traces in gray, mean in black)

### Brachial nerve recordings reveal a population of disproportionately represented, low-threshold, predominantly rapidly adapting digit-tip afferents

Single- and multi-unit recordings from the brachial nerve yielded 147 well-isolated mechanoreceptive units from 6 frogs (Fig. 3a). The distribution of receptive field locations was strikingly non-uniform: 89 of 147 units (60.5%) had receptive fields located entirely or predominantly on the digit tip lobules, despite the lobules comprising only approximately 8% of the total forelimb skin surface area (Fig. 3b). The remaining units had receptive fields centered on the digit shafts (34 units, 23.1%), the palm (17 units, 11.6%), or the forearm (7 units, 4.8%). The disproportionate representation of digit tip units in our brachial-nerve recordings is consistent with elevated peripheral innervation of the digit tips, though we acknowledge that the proportional unit count is sensitive to electrode-sampling biases (e.g., toward larger-caliber fibers) and does not by itself establish a fiber count.

Digit tip units had significantly lower mechanical thresholds (median: 0.12 mN, IQR: 0.04–0.4 mN) than digit shaft units (median: 1.4 mN, IQR: 0.7–2.0 mN; Mann-Whitney *U* = 312, *p* < 0.001, *r* = 0.87). The majority of digit tip units (71/89, 79.8%) exhibited rapidly adapting (RA) responses, firing briskly at stimulus onset and offset but not during sustained indentation (Fig. 3c). The remaining 18 digit tip units (20.2%) exhibited slowly adapting (SA) responses, with sustained firing that decreased gradually over the 5-s stimulus period. Digit shaft and palm units were more evenly divided between RA (51.0%) and SA (49.0%) response types.

Receptive fields of digit tip RA units were notably small: mean area = 0.31 ± 0.14 mm^2^ (range: 0.08–0.72 mm^2^), often confined to one or two secondary lobules. Digit tip SA units had larger receptive fields (mean: 0.88 ± 0.36 mm^2^; *t*(87) = 8.9, *p* < 0.001, Cohen’s *d* = 2.3), typically spanning a full primary lobe or the junction between two lobes. For comparison, digit shaft units had receptive fields of 3.2 ± 1.8 mm^2^, and palm units 8.4 ± 3.6 mm^2^. The progressive decrease in receptive field size from palm to digit shaft to digit tip is consistent with an increasing density of innervation at the digit tips.

A subset of digit tip RA units (23/71, 32.4%) responded to laminar water flow directed across the digit tips (Fig. 3d). These flow-sensitive units responded to flow onset with a burst of spikes that adapted within 200–500 ms, consistent with the RA character of these afferents. Response thresholds ranged from 2 to 8 cm/s (median: 4 cm/s), and firing rate increased monotonically with flow velocity up to the maximum tested (15 cm/s). Digit shaft and palm units (0/58 tested) did not respond to water flow at any velocity tested. The flow sensitivity of digit tip RA units indicates that the digit tip lobes are capable of detecting hydrodynamic disturbances, a capacity consistent with a role in the detection of swimming prey at short range.

### Cobalt selectively silences lateral-line input without affecting digit-tip mechanoreceptors

Before drawing inferences about behavioral mechanism from the IR + cobalt experiment, we verified directly in *P. pipa* that 0.1 mM CoCl_2_ silences lateral-line afferent activity while leaving digit-tip mechanosensory afferents intact. In 2 adult animals, simultaneous recordings from a dorsal-trunk superficial-neuromast afferent and a brachial-nerve digit-tip afferent were obtained before, during, and after a 30 min bath application of 0.1 mM CoCl_2_ (Supplementary Fig. S1). Baseline lateral-line afferent firing (mean 8–18 spikes/s) fell to < 1 spike/s within 8–12 min of cobalt onset and remained suppressed for the duration of cobalt exposure; on washout, firing recovered to > 75% of baseline within 30 min. By contrast, brachial-nerve afferent responses to 0.4 mN von Frey indentation (mean spike count per stimulus) and to 6 cm/s laminar flow (mean burst count) were statistically indistinguishable across pre-cobalt, mid-cobalt, and post-cobalt periods (paired Wilcoxon, both *p* > 0.4).

### Digit tip lobes are disproportionately magnified in the contralateral optic tectum

**Fig. 4:**
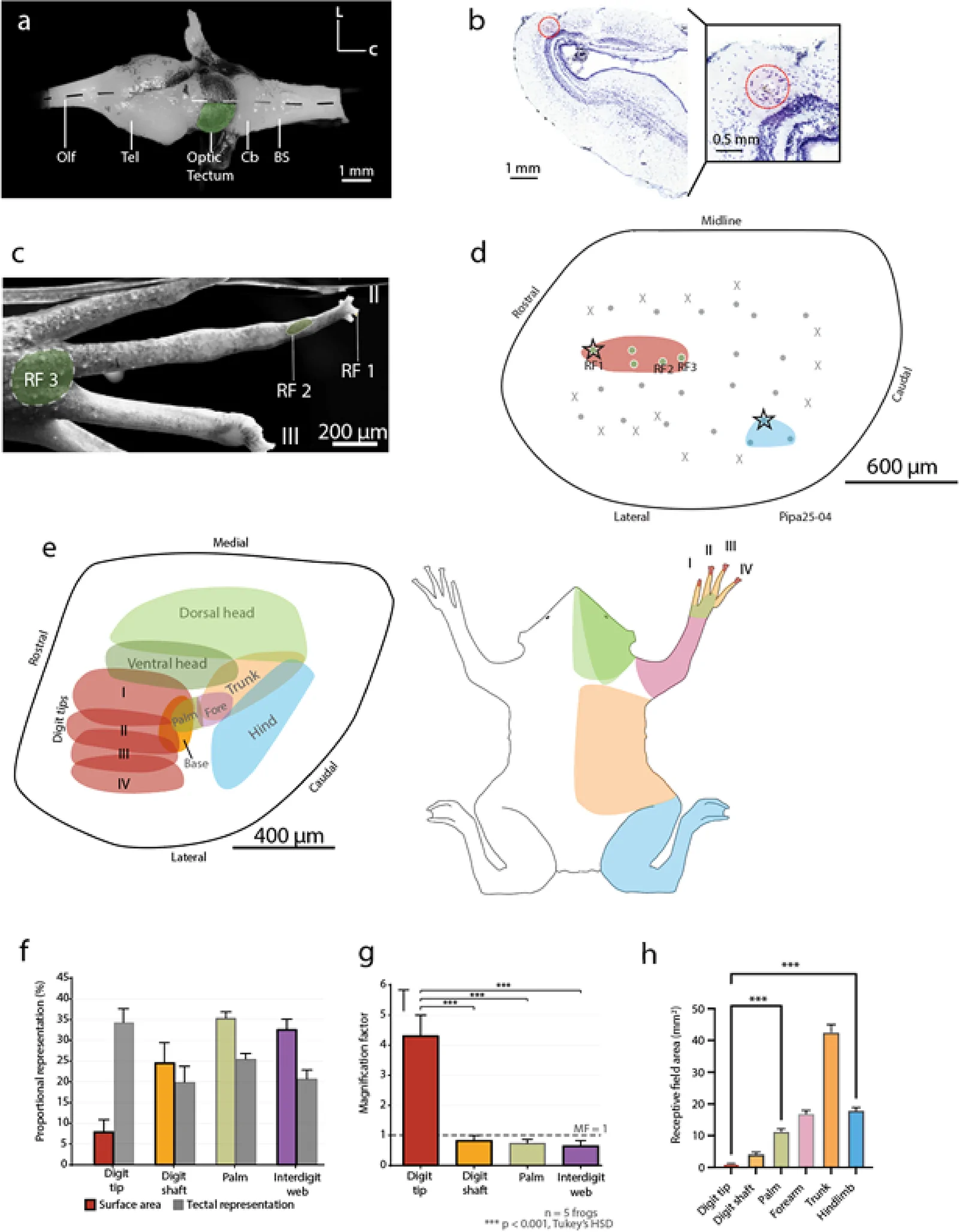
Tectal somatotopic mapping and magnification analysis. **a** Dorsal view of the *Pipa* brain showing the relative location of the optic tectum. *Olf* olfactory bulb, *Tel* telencephalon, *Cb* cerebellum, *BS* brainstem. The midline is indicated by a black dashed line. **b** Thionin-stained tangential section through the tectum with a higher magnification image of a lesioned point (inset). **c** Representative somatotopic mapping from one case. Three receptive fields (RF) are drawn, spanning areas on digit II and the palm. Scale bar 200 µm. **d** Corresponding schematic of the somatotopic mapping case showing electrode recording sites (gray circles), non-responsive areas (X), and sites of lesions (star), with areas responding to stimulation of the digit forelimb digit (red) and hindlimb (blue). **e** Tectal map of somatosensory unit responding to digit tip (red), digit shaft/base (dark yellow), palm (yellow/green), upper forelimb (magenta), dorsal surface of head (light green), ventral head (dark green), trunk (orange), and hindlimb (blue) stimulation. Left, schematic of dorsal view of *Pipa* following the same coloring conventions. **f** Comparison of proportional surface area of forelimb regions (left bars) versus proportional tectal representation (right bars), plotted as mean ± SD across n = 5 frogs. **g** Magnification factors across forelimb regions. **h** Comparative receptive field size as recorded in the contralateral tectum (n = 5 frogs; one-way ANOVA, ***p < 0.001 for digit tips vs. all other regions)

Systematic multi-unit mapping of the contralateral optic tectum in 5 frogs (216 responsive recording sites total) revealed a somatotopic representation of the forelimb in the caudolateral tectum (Fig. 4). Forelimb-responsive sites were found in the deep tectal layers (300–500 μm below the surface), consistent with the known laminar distribution of somatosensory inputs in the anuran tectum (Lázár 1984; Lowe 1986; Muñoz et al. 1995) (Fig. 4b). The overall somatotopic organization was orderly: medial tectal sites corresponded to upper body and head structures, while lateral sites corresponded to more ventral structures (digits, palm, forearm), and rostral-to-caudal tectal positions mapped onto distal to proximal forelimb positions.

Analysis of the proportional tectal area devoted to each forelimb region revealed a striking over-representation of the digit tip lobes (Fig. 4c–e). Across the 5 cases, the digit tips occupied a mean of 34.2 ± 4.1% of the total forelimb somatosensory representation in the tectum, despite comprising only 7.8 ± 0.6% of the total forelimb skin surface area (Fig. 4f). The magnification factor (proportional tectal area / proportional surface area) for the digit tips was 4.3 ± 0.7 (Fig. 4g). By contrast, the digit shafts had a magnification factor of 0.8 ± 0.2, the palm 0.5 ± 0.1, and the forearm 0.4 ± 0.1 (one-way ANOVA on magnification factors, *F*(3,16) = 38.7, *p* < 0.0001; Tukey’s HSD: digit tips vs. all other regions, *p* < 0.001; no other pairwise comparisons significant). The 4.3-fold magnification of the digit tips is comparable to the approximately 3–4-fold magnification of the 11th ray of the star-nosed mole in primary somatosensory cortex (Catania and Kaas 1997b; Catania and Remple 2004).

Receptive field sizes measured at tectal recording sites were smallest for digit tip sites (mean: 0.29 ± 0.11 mm^2^), intermediate for digit shaft sites (1.47 ± 0.52 mm^2^), and largest for palm and forearm sites (3.21 ± 1.03 mm^2^ and 5.8 ± 2.1 mm^2^, respectively; Kruskal-Wallis *H* = 68.3, *p* < 0.0001; Dunn’s post-hoc: all pairwise comparisons *p* < 0.01; Fig. 4h). The small tectal receptive fields for digit tip sites confirm that the magnified tectal representation supports higher spatial resolution at the digit tips than elsewhere on the forelimb. Together, the magnified representation and reduced receptive field size satisfy the second criterion for a somatosensory fovea.

### Prey capture is initiated before direct contact, even without vision or lateral line input

**Fig. 5:**
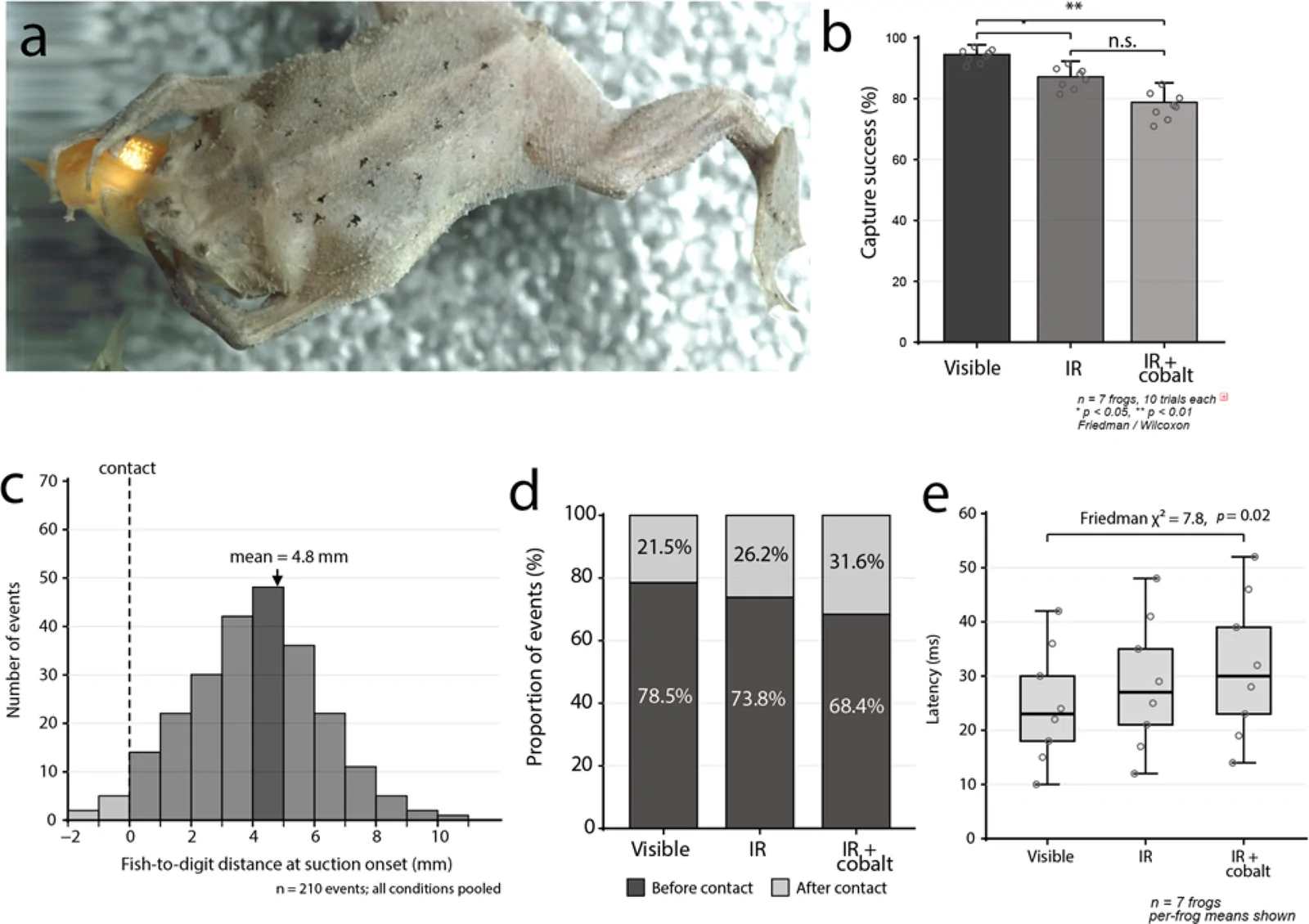
High-speed behavioral analysis of prey capture. (**a**) Frame of juvenile *Pipa* securing a live minnow under lighted conditions. Note the use of the forelimbs in capturing and manipulating the fish towards the mouth. (**b**) Capture success rates under three conditions (*n* = 7 frogs; Friedman’s test with post-hoc Wilcoxon, *p < 0.05, **p < 0.01). (**c**) Histogram of fish-to-digit distances at suction initiation across all conditions (*n* = 210 events). Dashed line at 0 mm indicates direct contact. (**d**) Proportion of events in which suction was initiated before (dark) or after (light) direct contact. (**e**) Latency to initiate suction once the fish has moved into the detection envelope, approximately 8 mm from a digit, across conditions

High-speed video analysis of 210 feeding events (7 frogs × 10 trials × 3 conditions) demonstrated that *P. pipa* captures prey with high success across all conditions (Fig. 5a). Mean capture success was 94.5 ± 3.2% under visible light, 87.2 ± 5.1% under IR-only, and 78.8 ± 6.4% under IR + cobalt (Friedman’s *χ*^2^ = 14.6, *p* < 0.001; post-hoc Wilcoxon: visible vs. IR, *p* = 0.04; visible vs. IR+cobalt, *p* = 0.008; IR vs. IR+cobalt, *p* = 0.06) (Fig. 5b). The maintenance of high capture success in the absence of both vision and lateral line function demonstrates that non-visual, non-lateral-line sensory modalities—most plausibly mechanosensory detection via the digit tips—are sufficient to guide prey capture.

Frame-by-frame analysis revealed that suction feeding was typically initiated when the prey fish was at a short but non-zero distance from the nearest digit surface (Fig. 5c). The mean fish-to-digit distance at suction initiation was 4.8 ± 1.9 mm across all conditions (visible: 5.1 ± 2.0 mm; IR: 4.6 ± 1.8 mm; IR+cobalt: 4.7 ± 2.1 mm; no significant difference among conditions, Friedman’s *χ*^2^ = 1.2, *p* = 0.55). Critically, in 73.2% of all feeding events (visible: 78.5%; IR: 73.8%; IR+cobalt: 68.4%), the prey fish had not yet made direct physical contact with any digit surface at the moment suction was initiated (Fig. 5d). Pre-contact suction-initiation proportions were 76.0 ± 4.2% (adults) and 71.1 ± 7.6% (juveniles) across conditions. Even in the IR + cobalt condition, where both vision and lateral line were eliminated, over two-thirds of suction feeding events were initiated before contact. This result indicates that the digit tips detect the approaching prey at a distance, most likely through the hydrodynamic pressure disturbance that precedes a swimming fish.

The latency between the prey fish’s first entry into the 8-mm detection envelope and suction initiation was 24.3 ± 8.7 ms under visible conditions, 28.6 ± 10.2 ms under IR, and 31.2 ± 11.4 ms under IR+cobalt (Fig. 5e). The modest increase in latency across conditions was significant (Friedman’s *χ*^2^ = 7.8, *p* = 0.02), suggesting that visual and lateral line inputs contribute to detection speed but are not required. These latencies are short, comparable to the 120-ms prey identification time of the star-nosed mole (Catania 2012), though the suction initiation latency reflects a different decision threshold (initiate suction vs. identify as edible). The short latencies and the high proportion of non-contact initiations together indicate a fast-acting, short-range mechanosensory detection system localized to the digit tips.

## Discussion

### The digit tip lobes are somatosensory foveae

**Fig. 6:**
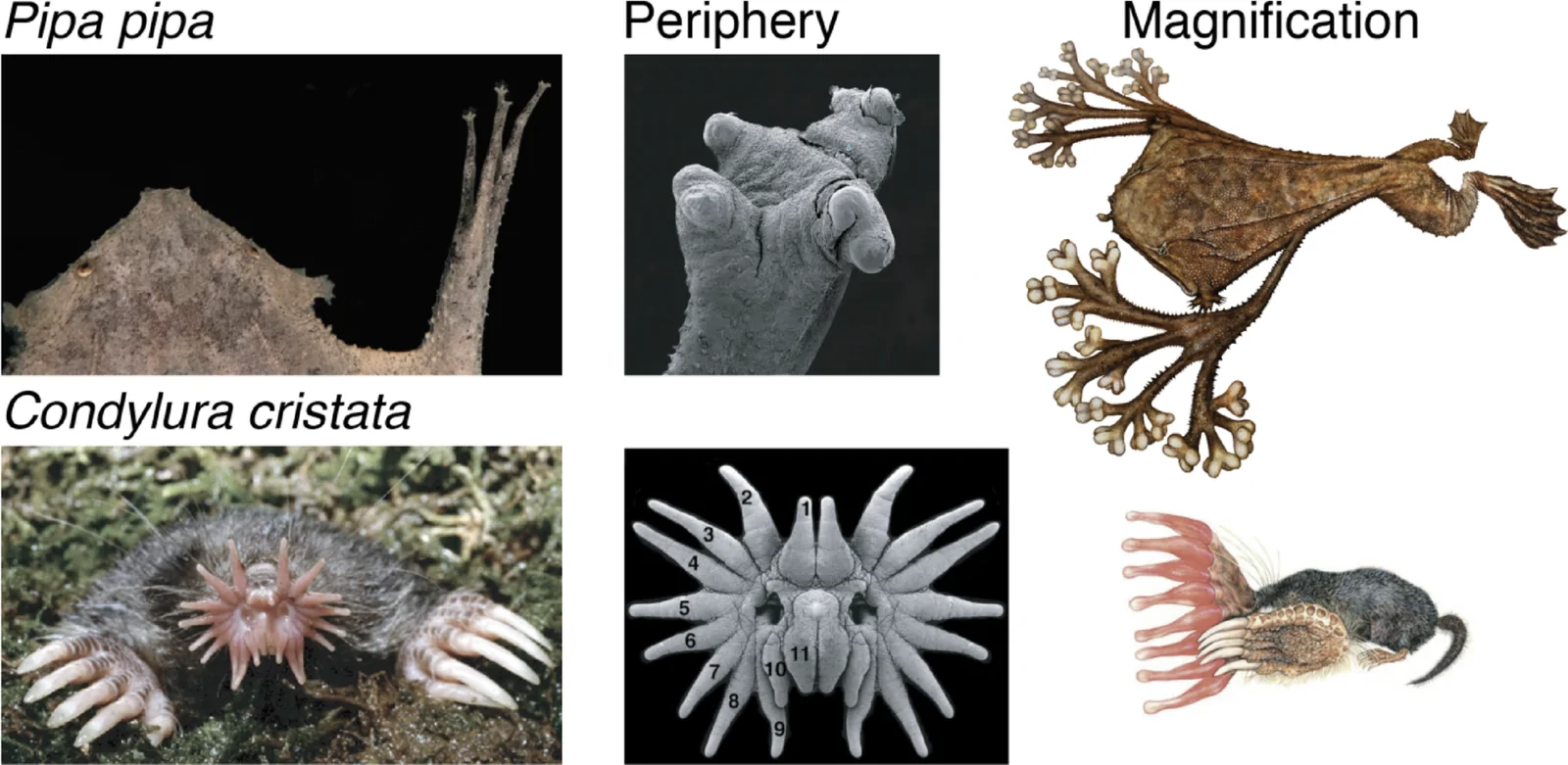
Summary: the digit tip somatosensory fovea of *Pipa pipa*. Top. Stereotypical ambush position of *Pipa* with forward-flexed forelimb, exposing digit tips. In adults, each digit tip is covered in four primary lobes which each quadrificate into four secondary lobes, totaling 128 structures per frog. These digit tips are disproportionately represented in the deeper layers of the contralateral tectum, as represented in the homunculus-style illustration (drawn to tectal magnification factors) with exaggerated forelimb and forelimb lobules. Illustration by author Rebeca Fuquen. Bottom. Convergence: this configuration in *Pipa* is reminiscent of the tactile magnification in the star-nosed mole (*Condylura cristata*) with its notable arrangement of 22 appendages surrounding the nares. At the level of primary somatosensory cortex (S1), the star representation occupies a disproportionately large area compared to the body surface, illustrated in this homunculus-style depiction of the mole, with exaggerated star and forelimbs. Bottom panels are used with permission (Catania 2005)

The framework that allows us to interpret these results, in which a specialized peripheral structure, an enlarged central representation, and a stereotyped sensorimotor program together constitute a functional sensory module, owes much to Peter Narins’s body of work on the sensory neuroethology of anurans (Narins and Capranica 1976; Feng et al. 2006; Narins et al. 2005), to whom this paper is dedicated. The results presented here establish the lobed digit tips of *Pipa pipa* as somatosensory foveae using the three-criterion framework developed by Catania and colleagues for the star-nosed mole (Pettigrew and Frost 1985; Catania and Kaas 1997b; Catania and Remple 2004) (Fig. 6). First, the digit tip lobules bear a density of dome-shaped papillae (142/mm^2^) nearly fourfold higher than the adjacent digit shaft (38/mm^2^), satisfying the elevated receptor density criterion. Second, the digit tips are disproportionately magnified in the contralateral optic tectum (magnification factor 4.3×, occupying 34% of the forelimb representation from 8% of the surface area), satisfying the central over-representation criterion. Third, the forward-flexed posture of the forelimbs during ambush, and the active repositioning of the hands during prey capture, constitute a sensorimotor strategy that positions the digit tips in the path of approaching prey, satisfying the behavioral repositioning criterion.

We have used the term “somatosensory fovea” in the strict three-criterion sense previously established by Catania and colleagues. We acknowledge, however, a legitimate semantic concern: on this definition, the primate hand, the human lips, and other regions of the cortical homunculus would also qualify, given that they show elevated receptor density, disproportionate cortical representation, and active sensorimotor repositioning. We view this as a feature of the framework rather than a problem with our usage, identifying a recurring organizational solution that evolution has converged on across modalities and lineages. We also note that the foveal concept can be applied hierarchically: in the star-nosed mole, the whole star is a fovea relative to the body, and appendage 11 is a fovea within the star. The *Pipa pipa* digit tips appear analogous to the whole-star fovea (i.e., the forelimb-vs.-body comparison); a finer subdivision (e.g., asymmetry across the four digits, or across the four primary lobes of a digit) would be the search target for a within-fovea “appendage 11” analog. Our data do not yet support such a within-fovea claim, but such digit-specific specialization remains an open question. Intriguingly, in the mammalian homolog to the tectum, the SC, the star-nosed mole’s 11th appendage is comparably represented to the other appendages of the star (which itself has a disproportionately large SC tactile representation) (Crish et al. 2003), a situation similar to our overall observations of relative uniformity among the exaggerated digit tip representations in the *Pipa* tectum.

The magnitude of tectal magnification we report (4.3-fold) is remarkably similar to the primary somatosensory (S1) cortical magnification of the 11th ray in the star-nosed mole, approximately 3–4-fold (Catania and Kaas 1997b; Catania and Remple 2004), the over-representation of the retinal fovea in primate V1, 3–5-fold depending on eccentricity and species (Azzopardi and Cowey 1993), and the expanded cortical representation of the acoustic fovea in the mustached bat (Suga et al. 1987). The convergence of magnification factors in this range, across sensory modalities and vertebrate classes separated by hundreds of millions of years of evolution, raises the possibility that a three- to five-fold magnification represents a sweet spot in the allocation of neural processing resources: sufficient to substantially enhance foveal resolution without excessively degrading the representation of the surround. This is, of course, speculative, with the optimal magnification factor depending on the dimensionality of the sensory surface, the distribution of behaviorally relevant stimuli, and the metabolic cost of neural tissue. We note, however, that foveal organization is not universal across sensory modalities—it is uncommon in the auditory system outside of FM-CF bats—suggesting that additional ecological and computational preconditions may be required. Nonetheless, the repeated convergence is suggestive of a general computational constraint. The convergence between *Pipa* and *Condylura* extends beyond the head/snout fovea analogy. In both species the forelimb is the behaviorally critical effector that delivers the foveal surface to prey: in the mole the powerful forelimbs are themselves enlarged in S1 and S2 (Catania and Kaas 1997b), and in *Pipa* the forelimb is the body region from which the entire prey-encounter sequence is initiated.

The magnification factor we report (4.3×) is by construction a ratio, and its numerical value depends on the choice of denominator. We computed it as the proportion of tectal area devoted to digit tips divided by the proportion of forelimb surface area occupied by digit tips. Computed against whole-body surface area the absolute value would be different (and would depend on the proportion of tectum devoted to the forelimb overall); computed against neighboring digit-shaft surface area it would be larger still. We chose the forelimb-as-denominator framing because it parallels previously star-nosed mole measurements (Catania and Kaas 1997b) which computed the 11th appendage’s magnification against the whole-star area. Across reasonable choices of denominator the qualitative conclusion is unchanged: digit-tip lobules occupy a fraction of the tectum that vastly exceeds their fraction of the relevant skin surface.

To check whether the pooling of 2 adults and 3 juveniles obscured any age-dependent differences, we computed group means separately. Magnification factors for digit tips were 4.5 ± 0.6 in adults and 4.2 ± 0.8 in juveniles (Welch *t* = 0.51, df = 2.8, *p* = 0.65); proportional tectal representation was 35.1 ± 3.0% in adults vs. 33.6 ± 5.0% in juveniles. Although power is low, the qualitative pattern is the same in both groups, consistent with the SEM observation that primary quadrification (and thus most of the lobule peripheral structure) is well developed by the juvenile stage even though secondary lobules are partially fused.

### Peripheral mechanoreceptive specialization of the digit tips

The electrophysiological properties of digit tip afferents closely parallel those of mechanoreceptive afferents described in other high-acuity somatosensory systems. The predominance of rapidly adapting units (80% of digit tip afferents) with small receptive fields (mean 0.31 mm^2^) is reminiscent of the RA afferents innervating Meissner corpuscles in primate fingertips (Johansson and Vallbo 1979) and the primary afferents supplying Eimer’s organs in the star-nosed mole (Marasco and Catania 2007). In all three systems, the predominance of RA afferents suggests optimization for detecting dynamic mechanical events (the onset and offset of contact, or changes in pressure) rather than sustained force. For *Pipa*, this tuning is functionally appropriate for detecting the transient hydrodynamic disturbances generated by a swimming fish, which consist of brief, dynamic pressure fluctuations rather than steady-state flow (Bleckmann 1994; McHenry et al. 2008).

The lobule tip thresholds measured here (median 0.07 mN) are among the lowest reported for any amphibian mechanoreceptor. For comparison, the thresholds of crocodilian ISOs on the distal digits, among the most sensitive mechanoreceptors described for any vertebrate, are approximately 0.08–0.4 mN (Leitch and Catania 2012). Primate fingertip Meissner corpuscle thresholds are in the range of 0.03–0.1 mN (Johansson and Vallbo 1979). The sensitivity of the *P. pipa* digit tip lobes thus approaches that of the most refined mammalian and reptilian touch organs.

The sensitivity of a subset of digit tip RA afferents to laminar water flow (threshold ~ 4 cm/s) is a noteworthy finding that distinguishes the *Pipa* digit tip system from the somatosensory fovea of the star-nosed mole, which operates in a substrate-contact mode (Catania and Remple 2004). The flow-sensitive digit tip afferents are functionally intermediate between the contact-based mechanoreceptors of terrestrial somatosensory foveae and the displacement-sensitive hair cells of the lateral line system. This dual sensitivity to both direct contact and water-borne hydrodynamic stimuli may explain the remarkable ability of *Pipa* to detect prey at a distance (mean ~ 5 mm) in the absence of both vision and lateral line function. A swimming minnow of the size used in our experiments (15–25 mm) generates a bow wave and wake flow of 1–10 cm/s at distances of 3–8 mm from the body (Bleckmann 1994), well within the sensitivity range of the flow-responsive digit tip afferents. For comparison, anuran lateral-line superficial neuromasts in adult *Xenopus* have flow-velocity thresholds in the range of 0.03–1 mm/s (Kroese et al. 1978; Strelioff and Honrubia 1978), one to two orders of magnitude more sensitive than the digit-tip flow-responsive afferents described here (threshold ~ 4 cm/s). The digit-tip flow channel is thus less sensitive than the lateral line per se but operates at very short range and with high spatial resolution, and complements rather than duplicates lateral-line function.

The dome-shaped papillae on the digit tip lobules share some morphological features with the integumentary sensory organs (ISOs) of crocodilians (von Düring and Miller 1979; Soares 2002; Leitch and Catania 2012), including a raised dome profile, thin overlying epidermis, and dense innervation. Crocodilian ISOs function as exquisitely sensitive mechanoreceptors capable of detecting surface water ripples generated by prey, with thresholds (0.08–0.4 mN) comparable to those we report here for *Pipa* digit tip lobules (0.07 mN). The functional parallel is striking: both crocodilians and *Pipa* are aquatic ambush predators that use dome-shaped integumentary mechanoreceptors to detect prey-generated water disturbances, and in both cases the most sensitive organs are concentrated on the surfaces that first encounter prey during capture (the jaws and digit tips, respectively). Whether the papillae of *Pipa* and crocodilian ISOs share homologous receptor cell types (e.g., Merkel cell–neurite complexes, lamellated corpuscles) or represent convergent solutions assembled from different cellular components is a question for future histological and molecular investigation.

Our cobalt control experiment confirms that 0.1 mM CoCl_2_ selectively silences lateral-line afferent activity while leaving digit-tip mechanosensory afferents intact (Supplementary Fig. S1). These data validate the IR + cobalt behavioral condition as eliminating lateral-line function without compromising the digit-tip somatosensory channel.

### Could the ear contribute?

Anuran otolithic endorgans, particularly the saccule and lagena, are sensitive to near-field hydrodynamic disturbances and substrate vibration (Christensen-Dalsgaard and Narins 1993; Braun and Sand 2013), and in principle could detect the pressure perturbation generated by a swimming fish at the short ranges over which we observe pre-contact suction initiation. Three lines of evidence argue that an auditory/otolithic channel is unlikely to be the dominant contributor to the behavior we describe. First, the tectal receptive fields for pre-capture-relevant sites are small and localized to individual secondary lobules; saccular input alone would not be expected to produce such finely localized, digit-specific receptive fields. Second, the detection envelope is centered on the digit tips, not on the head, with suction onset proportions depending on which specific digit-tip surface the fish has approached. Third, the strong correlation between mechanical thresholds and SEM-defined papillae density (Spearman *ρ* = −0.91, *p* < 0.001) and the tectal magnification gradient that peaks at the same lobule surfaces anchor the digit-tip mechanosensory interpretation independently of any auditory contribution. Nonetheless, we cannot exclude a contributory role for the saccule, and the cleanest experiment would be a saccular or VIIIth-nerve manipulation under IR + cobalt + intact-otolith versus IR + cobalt + otolith-ablated comparison. We propose this as the natural next step.

### Chemosensory possibilities

Because our IR + cobalt condition does not isolate somatosensation from cutaneous chemosensation, we address this alternative explicitly. We have not selectively eliminated chemosensation in our behavioral assays, and the IR + cobalt condition therefore leaves both somatosensory and any cutaneous chemosensory channels intact. Solitary chemosensory cells and ionocyte-like elements have been described in the skin of *Xenopus laevis* (Whitear 1992; Quinzio and Reiss 2018), and in larval and aquatic amphibians more broadly, cutaneous salinity- and amino-acid sensitivity is documented. We cannot exclude a cutaneous chemosensory contribution to prey detection in *Pipa*. However, the very short detection-to-suction latencies we observe (24–31 ms) are difficult to reconcile with a primary chemosensory role, given the diffusion-limited time courses of cutaneous chemodetection; and the spatial precision of the prey-localization behavior is more consistent with mechanosensory than chemosensory mechanisms. Targeted chemosensory blockade experiments (e.g., topical amiloride) are an important future direction.

### Somatosensory foveal magnification in the anuran tectum

Prior demonstrations of central magnification for somatosensory foveae have come predominantly from mammals (Catania and Kaas 1997b; Catania 2011; Sawyer et al. 2014), with peripheral and behavioral evidence (but not direct central-magnification demonstration) in shorebirds (Pettigrew and Frost 1985) and the platypus (Pettigrew 1999). Our demonstration that foveal magnification occurs in the amphibian optic tectum extends this principle to a fundamentally different neural architecture. Amphibians lack a neocortex entirely; the pallial derivatives of the amphibian telencephalon are simple, laminar structures that do not receive direct somatosensory thalamocortical projections in the manner of mammalian S1 (Roth 1987). Instead, spatial sensory processing and sensorimotor integration in anurans are centered in the optic tectum, a midbrain structure homologous to the mammalian SC (Ewert 1970; Lázár 1984).

The tectum is a multi-layered structure that receives topographically organized inputs from the retina (in superficial layers), from the auditory system, and from the somatosensory system (in deep layers), all in spatial register (Lázár 1984). In anurans, the tectum plays a central role in prey-capture behavior: visually guided tongue strikes in ranid frogs are mediated by tectal circuits that transform retinotopic spatial information into motor commands directed at the prey’s location (Ewert 2004). Our data suggest that in *Pipa*, the deep-layer somatotopic map of the digit tips may serve an analogous function, transforming the spatial location of a tactile or hydrodynamic stimulus on the digit surface into a motor command for suction feeding. The very short latencies we observe between prey detection and suction initiation (24–31 ms) are consistent with a monosynaptic or oligosynaptic tectofugal motor pathway, similar to the fast tectal circuits underlying visually evoked prey capture in other anurans (Ewert 2004).

The finding that somatosensory foveal magnification can be implemented in the tectum has a broader implication: it suggests that the principle of foveal magnification is not tied to a specific brain structure (neocortex vs. tectum) but is a general feature of how neural tissue is allocated to behaviorally important sensory inputs. This is consistent with the view that foveal magnification is driven primarily by the differential activity of sensory afferents during development and by the competitive dynamics of activity-dependent circuit refinement (Merzenich and Jenkins 1993; Catania 2001), processes that operate in the tectum as well as in the neocortex.

### Relation to the torus semicircularis

The torus semicircularis is primarily the auditory midbrain nucleus in anurans, though its lateral subdivision has been reported to receive somatosensory input with predominantly broad receptive fields (Comer and Grobstein 1981a, b; Muñoz et al. 1995, 1997). The small, finely localized receptive fields we describe in the deep optic tectum (mean 0.29 mm^2^ for digit-tip sites) thus appear refined relative to the receptive-field properties classically reported for the lateral torus. Three considerations argue that our recordings are genuinely from deep optic tectum rather than from torus. First, recording depth (200–500 μm below the tectal surface) within the laminar location of histologically verified electrolytic lesions (Fig. 4b) place the recording sites within the deep layers of the tectum proper (Wilczynski and Northcutt 1977). Second, the rostrocaudal/mediolateral somatotopic organization we observe is consistent with tectal organization rather than with the more limited topography reported for torus. Third, deep-tectal somatosensory inputs have been described in anurans (Lowe 1986; Muñoz et al. 1995). We do not, however, claim that the tectum is the sole midbrain somatosensory recipient in *Pipa*; the torus may also carry forelimb input and could contribute parallel processing. Parallel mapping of the torus in *Pipa* is an important target for future work.

### Ecological and phylogenetic context

The evolution of the digit tip lobes in *Pipa* must be understood in the context of the sensory challenges faced by a tongueless, ambush-predatory frog in turbid, visually impoverished aquatic habitats. *P. pipa* inhabits warm, acidic, slow-moving waters with high concentrations of dissolved organic matter and tannins, resulting in visibility that may be limited to a few centimeters (Rabb and Rabb 1963). Its eyes are small, dorsally directed, and lidless, providing at best coarse overhead predator detection rather than precise lateral prey localization. Unlike most anurans, which capture prey using visually guided ballistic tongue projection, *P. pipa* must rely on non-visual modalities to detect and localize prey at close range in order to initiate its rapid suction feeding strike.

Our data demonstrate that the digit tip lobes can fill this sensory niche. They provide high-resolution mechanosensory information about the location and movement of nearby prey, with sufficient sensitivity to detect the hydrodynamic signature of an approaching fish before direct contact. This capacity appears to be a derived specialization within Pipidae, given that the degree of digit tip lobing varies across the family: *Xenopus* (which inhabits somewhat clearer waters and is a more generalized feeder) has simple digit tips, while *P. pipa* (which is the most specialized suction feeder and inhabits the most turbid environments) has the most elaborate tips. This phylogenetic gradient is consistent with the hypothesis that digit tip elaboration was driven by selection for enhanced mechanosensory prey detection in an aquatic, visually constrained environment. We note, however, that we have not performed a formal phylogenetic comparative analysis, and additional functional data from other pipid species would be needed to rigorously test this hypothesis.

It is also worth considering the digit tip lobes in the broader context of mechanosensory systems used by aquatic vertebrates for prey detection. Fish and larval amphibians use the lateral line system, which consists of superficial and canal neuromasts innervated by cranial nerve afferents (Bleckmann and Zelick 2009). Crocodilians use ISOs on the jaws and face, innervated by the trigeminal nerve (Leitch and Catania 2012). The platypus uses electro- and mechanoreceptors on the bill (Pettigrew 1999). In each case, the sensory organ is positioned on the body surface that first encounters prey during the capture sequence. The digit tip lobes of *P. pipa* conform to this pattern: they are positioned on the structure that leads the approach to prey and that flanks the buccal cavity during suction feeding. The convergent deployment of mechanosensory foveae on prey-contact surfaces across diverse aquatic vertebrate lineages underscores the general adaptive value of concentrating sensory resources at the point of prey contact.

### The somatosensory fovea as a general evolutionary solution

The independent evolution of somatosensory foveae in the star-nosed mole (a mammal), *P. pipa* (an amphibian), and possibly in the bill tips of probing shorebirds and crocodilian jaws suggests that foveal organization is a general evolutionary solution to the problem of constructing a high-resolution sensory system within the constraints of finite neural tissue. Rather than investing in high receptor density and high-resolution central representation across the entire sensory surface, which would impose prohibitive metabolic and volumetric costs, evolution favors concentration of resources at a small, behaviorally critical region, coupled with active sensorimotor behavior that repositions this region onto stimuli of interest. This strategy is immediately recognizable from the visual system (Yarbus 1967; Land and Nilsson 2012).

The *Pipa* system offers unique advantages as a model for future investigation of somatosensory foveal organization. Unlike the star-nosed mole, which is a fossorial mammal that is difficult to maintain and breed in laboratory settings, *Pipa* is readily maintained in aquaria, breeds in captivity, and has emerging genomic resources (as part of the *Xenopus* genomics community’s comparative efforts). Its phylogenetic position as a basal anuran, within a family that includes the major model organism *Xenopus laevis*, provides a rich comparative framework for investigating the developmental, genetic, and neural mechanisms underlying the evolution of somatosensory foveae. The digit tip lobes of *Pipa* may therefore represent a powerful model for understanding how sensory foveae arise, develop, and are encoded in the central nervous system.

## Supplementary Information

Below is the link to the electronic supplementary material.

Supplementary Material 1. Supplementary Video S1 Visible-light prey capture, 1000 fps. In response to the approach of a moving fish towards their outstretched forelimbs and digits, *Pipa* initiated suction feeding, followed by forelimb movement to secure and guide prey into the mouth

Supplementary Material 2. Supplementary Fig. S1 Cobalt chloride efficacy control. a. Neural responses from a brachial nerve branch innervating lateral line neuromasts indicating the firing frequency (top), and individual spikes across conditions (baselines, mid-cobalt treatment, and following cobalt washout) (bottom). b. Neural responses from a brachial branch innervating digit tip. c. Peristimulus time histograms from brachial nerve branch innervating the digit tip in response to von Frey filament deflection and laminar flow. Responses are shown across cobalt treatment conditions (baseline, treated, and following wash-out). In response to direct mechanical (von Frey) or laminar flow, there is little change to spike frequency

## Data Availability

All data supporting the findings of this study are available from the corresponding author upon reasonable request.
