# Supplementary material for: A somatosensory fovea in the fingertips of the Surinam toad
Source: J Comp Physiol A Neuroethol Sens Neural Behav Physiol. 2026 Aug 10;212(5):965–84. doi: 10.1007/s00359-026-01838-w (PMC13642415; doi:10.1007/s00359-026-01838-w)
Supplement: Supplementary file 1 [file 359_2026_1838_MOESM2_ESM.pdf]

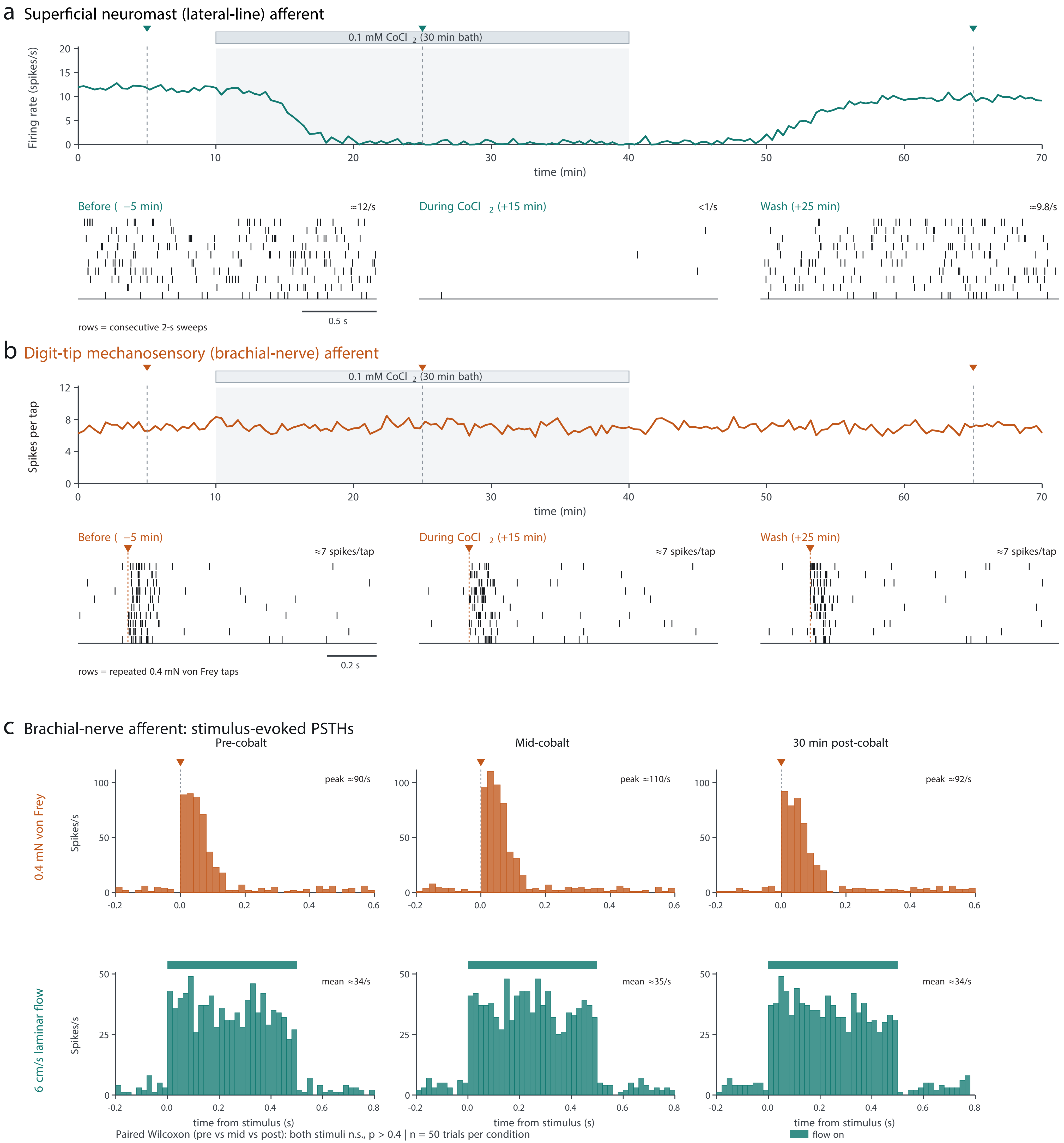

a Superficial neuromast (lateral-line) afferent
0.1 mM CoCl 2 (30 min bath)
Firing rate (spikes/s)
time (min)
Before (−5 min)
≈12/s
During CoCl 2 (+15 min)
<1/s
Wash (+25 min)
≈9.8/s
0.5 s
rows = consecutive 2-s sweeps
b Digit-tip mechanosensory (brachial-nerve) afferent
0.1 mM CoCl 2 (30 min bath)
Spikes per tap
time (min)
Before (−5 min)
≈7 spikes/tap
During CoCl 2 (+15 min)
≈7 spikes/tap
Wash (+25 min)
≈7 spikes/tap
0.2 s
rows = repeated 0.4 mN von Frey taps
c Brachial-nerve afferent: stimulus-evoked PSTHs
Pre-cobalt
Mid-cobalt
30 min post-cobalt
0.4 mN von Frey
Spikes/s
peak ≈90/s
peak ≈110/s
peak ≈92/s
6 cm/s laminar flow
mean ≈34/s
mean ≈35/s
mean ≈34/s
time from stimulus (s)
Paired Wilcoxon (pre vs mid vs post): both stimuli n.s., p > 0.4 | n = 50 trials per condition
flow on
